# Health Systems and Their Assessment: A Methodological Proposal of the Synthetic Outcome Measure

**DOI:** 10.3389/fpubh.2018.00126

**Published:** 2018-05-07

**Authors:** Piotr Romaniuk, Krzysztof Kaczmarek, Magdalena Syrkiewicz-Świtała, Tomasz Holecki, Adam R. Szromek

**Affiliations:** ^1^Department of Health Policy, School of Public Health, Medical University of Silesia in Katowice, Katowice, Poland; ^2^Department of Health Economics and Management, School of Public Health, Medical University of Silesia in Katowice, Katowice, Poland; ^3^Department of Computer Science and Econometrics, Faculty of Organization and Management, Silesian University of Technology, Gliwice, Poland

**Keywords:** health system, health system outcomes, health system assessment, health system performance, Central and Eastern Europe

## Abstract

The effectiveness of health systems is an area of constant interest for public health researchers and practitioners. The varied approach to effectiveness itself has resulted in numerous methodological proposals related to its measurement. The limitations of the currently used methods lead to a constant search for better tools for the assessment of health systems. This article shows the possibilities of using the health system synthetic outcome measure (SOM) for this purpose. It is an original tool using 41 indicators referring to the epidemiological situation, health behaviors, and factors related to the health-care system, which allows a relatively quick and easy assessment of the health system in terms of its effectiveness. Construction of the measure of health system functioning in such a way allowed its presentation in dynamic perspective, i.e., assessing not only the health system itself in a given moment of time but also changes in the value of the effectiveness measures. In order to demonstrate the cognitive value of the SOM, the analysis of the effectiveness of health systems in 21 countries of Central and Eastern Europe during the transformation period was carried out. The mean SOM values calculated on the basis of the component measures allowed to differentiate countries in terms of the effectiveness of their health systems. Considering the whole period, a similar level of health system effects can be observed in Slovenia, Croatia, Czech Republic, Slovakia, Poland, Macedonia, and Albania. In the middle group, Hungary, Romania, Latvia, Lithuania, Georgia, Estonia, Bulgaria, Belarus, and Armenia were found. The third group, weakest in terms of achieved effects, was formed by health systems in countries like Ukraine, Moldova, and Russia. The presented method allows for the analysis of the health system outcomes from a comparative angle, eliminating arbitrariness of pinpointing a model solution as a potential reference point in the assessment of the systems. The measure, with the use of additional statistical tools to establish correlations with elements of the external and internal environment of a health system, allows for conducting analyses of conditions for differences in the effects of health system operation and circumstances for the effectiveness of reform processes.

## Introduction

Among terms that are frequently exploited in public health and health politics literature, the notions most willingly used are “health-care system” and “health system.” These terms become a subject of many debates coming down to a question of whether they are synonymous or different. Not resolving the dilemma, it is worth noting that in both cases, there is a consensus as to perceiving them as a system. Following Arrington and Kurz, it can be stated that a system is a certain wholeness—a set of co-dependent elements (people, processes, products, and services) which are connected by a common purpose (1). Transferring this understanding onto health ground, it is possible to define a health system as an institutional configuration encompassing all entities which realize a health goal within the range of their activities and interactions. In this publication, the authors have employed the term “health system,” considering it as broader in terms of range and more adherent to the chosen empirical purposes. Simultaneously, it deserves to be stressed that the analyzed system is characterized by a high level of openness, which has a direct effect on a large number of circumstances and interactions it is facing, as well as forming (2).

Health system on an analytical level most often exists in plural as the entirety of solutions used or existing in given countries, or less frequently, regions, and local environments. Being aware of differences in health system forms in various countries, a willingness to compare them in numerous aspects is to be considered naturally. Undoubtedly, one of the most important and often analyzed issues is the effectiveness of health systems. Leading a relevant discussion on the abovementioned topic requires at first defining the term “effectiveness” on which there is no unequivocal agreement in literature (3).

In the most basic sense, and, at the same time, closest to the popular understanding, effectiveness is to a large extent identical with the notion of efficiency, efficacy, or productivity. Holstein-Beck, reviewing the twentieth-century theories of effectiveness, discerned six basic notion categories constituting the elements of effectiveness. These are efficiency, productivity, competence, functionality, comprehensibility, and morality (3). The assumption of such a perspective means assessing as effective such activities which lead to maximization of the achieved goals. This, however, evades the aspect of input rationality, which can be perceived as a substantial flaw. At the same time, including the factor of relationship between the output and the input, the notion of effectiveness will become closer to the term “economicalness” (4).

Differences in perceiving effectiveness are also mirrored in the interpretation of effectiveness of health systems, leading to many separate proposals. The most frequently mentioned attitude in reference to the subject matter is the concept elaborated by the World Health Organization, according to which the measurement of effectiveness of a health system is made mainly through the outcome understood as productivity of health services provided to their receivers. In this approach, the authors have chosen altogether five measures matching the following dimensions: health, health inequalities, suitability (*responsiveness level* which can be understood as compliance of the health system functioning with its beneficiaries’ expectations, especially in non-health effects, thus, for instance, equal and adequate treatment, satisfaction, etc.), proportionality of adequateness (*responsiveness distribution* which in turn refers to evenness of satisfaction from health system operation, in particular in terms of non-health effects in various social groups), and fairness (*fair-financing* which pertains to financial aspects, particularly with regard to evenness of financial burdens among the given social groups, provided that a fair system should be constructed in a way which leads to charging beneficiaries from different social groups with the costs in the form of stable proportion to their obtained incomes) (5).

The approach suggested by the World Health Organization constitutes a combination of measures of objective (epidemiological data) and subjective nature (assessment of the patients’ satisfaction in health system operation). It also contains factors pertaining to the system productivity in the epidemiological understanding (improvement of the population’s health condition), as well as elements stemming from doctrinal premises. In this case, the doctrine framework holds that one of the aims of a health system is not only to improve health status of the population as a whole but to achieve this result in a balanced way and with the proportionality of financial burdens referring to various social groups. Finally, the aims of a health system in this approach are of multidimensional character pertaining to both health matters and a more "soft" area of the relationship system-to-patient, whose derivatives are the patient’s opinion on the system functioning, satisfaction level, and impression of finding a proper place for oneself in the system.

The WHO approach is nonetheless frequently an object of criticism, pinpointing such aspects as incomparability of systemic circumstances accompanying service realization in different countries, arbitrariness in the application of weights to given measures, and the instrumental treatment of financial aspects in which the relationship between the input and the output becomes of secondary value (6).

A separate point needs to be made about the problem of basing the assessment on factors outside the system and methodological difficulty in the necessity of conducting regular patients’ opinion poll, resulting from measurement construction.

Another approach to the measurement of health system effectiveness is offered by the Organization for Economic Co-operation and Development. OECD identified three possible levels for the analysis of the system effectiveness: health perspective (*disease*), subsystem area referring to functional aspects of entities providing health services, and systemic perspective, as thorough as possible.

The measurement of effectiveness in this approach will focus on a triangle encompassing entry factors (*inputs*—including staff and infrastructure of a system, as well as health-care expenditures) and *outcomes—*referring to the availability of health services measured by a number of medical visits, specialist consultations, or hospital procedures, thus pertaining to the epidemiological aspect and the so-called subsystem (7).

In addition to the two approaches mentioned above, literature provides a number of other methodologies for the measurement of effectiveness in health care. Some among them approach it from a technical angle, by means of DEA method (8). An example of such an approach is the work by I. Laskowska and K. Lewandowska (9), V.N. Bhat (10), J. Magnussen (11), or J. Rój (12). A wide overview of effectiveness issues in health care was presented in Polish literature by M.E. Kruk and L.P. Freedman (13), as well as Niemczyk (14). The latest available works on such issues are those by I. Papanicolas and P.C. Smith (15), J. Gerring et al. (16), as well as a publication by the Canadian Institute for Health Information (17).

A high number of existing methodologies are a result of different circumstances accompanying the investigated phenomenon, which forces researchers to seek alternative solutions. Repeatedly, a choice of measuring method or measurement factors is conditioned not only on their diagnostic or informative value but rather depends on pragmatism compelling rejection of solutions that would be too demanding or impossible to implement. As a result, a problem can occur of the impossibility to fully shift the existing methodology to the present conditions differing from those in which it was elaborated. It may be related to many problems: lack of credible data, mutual incompatibility of data or measurement criteria, or too far-stretched arbitrariness (necessary to some extent) in setting points of reference during measurement process.

In the above circumstances, we decided to apply authorial methodology, focusing on measuring the outcomes of the health system. Thus, we resign measuring the effectiveness, meaning a specific relationship between the effects and the input, although our methodological proposal is largely related to the same analytical areas as the concepts presented above. The reason for withdrawing the semantical framework of effectiveness was that our idea was to use a measurement method that gives the opportunity to analyze the relationship between the health system outcomes and the diverse elements characterizing its environment, including the amount of expenditures incurred on health care. It gives the opportunity to statistically analyze the impact of economic and organizational factors on the outcomes, and at the same time, it can become the basis for analyzing effectiveness in the strict sense, after applying additional analytical tools.

This paper aims at presenting a proposal for the synthetic measure of health system outcomes, potentially useful in making comparative analyses of health systems in terms of their performance, along with an example of its application in post-communist countries.

## The Method

### Definitions

Due to the ambiguity of the terminology relevant in context of the matter of this paper, Table 1 presents the key terms used in its content, along with the determination of their semantic scope.

**Table 1:** Basic terminology relevant in context of the presented methodological proposal.

Term	Definition
Health system	An institutional configuration, which include all bodies and institutions, that within their activities and interactions pursue a health goal. As such, the meaning of the term is broader than, i.e., “health care system,” which would refer only to those institutions, which are directly involved in the provision of health services.
Effectiveness	The ability to maximize feasibility of the assumed goals, within the framework of rational level of resources incurred to achieve these goals.
Health system outcomes	The result of the functioning of health system assessed by means of objective measures referring to the health status of the population, as well as the productivity of the health system in terms of modifying health behaviors and maintaining adequate infrastructure to achieve the health goal.

## Synthetic Outcome Measure (SOM)

The index of SOM was elaborated as an authorial proposal taking into account a number of premises and construction assumptions.

Firstly, we made an effort to keep compliance on a general level with theoretical approaches presented by subject literature, with particular emphasis on the WHO and OECD ones. The suggested approach differs from the aforementioned at a detailed level in reference to the elements used for a constructed measure and qualification of certain measures (like the subsystem defined by the OECD) which were not treated as parts of the measure of health system functioning but rather as potential determinants of its level.

We also assumed multidimensionality of understanding the effects of health system functioning which encompasses both an epidemiological level—treated with priority—and a level of medical service availability. In addition, the catalog of constituents was broadened by measures substituting the assessment of public health-care efficacy (measures describing a selected catalog of health behaviors) and relating to selected constituents of restructuring processes in health care.

Another assumption related to the proposed methodology was to ensure availability and easy access to data, provided that a maximum data completeness is granted in reference to every country of the study and data comparability, which was connected with harmonization of sources. Thanks to this assumption, the analysis of effects and assessment of the whole system are possible at every moment in a relatively fast and convenient way.

Finally, we aimed to construct the measure of health system functioning in such a way to allow its presentation in a dynamic perspective, i.e., assessing not only the health system itself in a given moment of time but also changes in the value of the effectiveness measures.

Based on the aforementioned assumptions, a catalog of the SOM index constituents was developed, involving measures referred to epidemiological result, result in terms of health behaviors which was accepted as a substitute for the efficiency of public health policy in a given state, result in a systemic area referring to the availability of health services, and dynamics of restructuring processes with the assumption of similar starting circumstances of extensive and inadequate structure of health system resources.

In addition, on the border of the epidemiologic result areas and health behaviors appeared the measures referring to the prevalence of cancer, diabetes, tuberculosis in the population, and mortality caused by external reasons. Due to the fact that health systems undergoing an intensive transformation process were assessed with the use of this method, constituents of the measure were used both in nominal values and in dynamic perspective, i.e., involving change in value of a given measure. A comprehensive summary of measures used to construct the index is presented in Table 2.

**Table 2:** Summary of measures constituting the SOM index.

Epidemiological result	Result in terms of health behaviors	Result in systemic terms
Life expectancy, mortality caused by contagious and parasitic diseases, mortality caused by diabetes, mortality caused by cardiovascular diseases (for general population and age group of 0–64), infant mortality, maternal mortality, cancer mortality, tuberculosis mortality	Alcohol intake per person, number of people smoking regularly, consumption of tobacco products (number of cigarettes smoked per person), number of accidents at work per 1,000 employees, percentage of deadly accidents at work	Yearly number of outpatient medical visits per person, number of hospital beds per 100,000 inhabitants, number of beds occupied for short hospitalization per 100,000 inhabitants, share of acute beds occupied in general number of hospital beds, level of hospital beds use, average hospitalization time

Prevalence of cancer diseases in population, incidence of tuberculosis, prevalence of diabetes in population, mortality for external reasons		

The use of the above collection of indicators leads to the selection of a set of variables of diversified nature. Boosters (stimulants), which growing, improved the assessment of the analyzed phenomenon, and inhibitors (destimulants), which growing in time, lowered the assessment of the phenomenon, as well as nominants, which, to a certain degree, behaved like boosters, and from a certain degree were treated as inhibitors. To estimate the SOM value, taxonomic methods were applied, including both harmonization of variables denomination, and their aggregation and weighting. An algorithm for the creation of the SOM coefficient was used with uniformization and normalization of variables which preceded weighting and aggregation of partial variables (18). Normalization was conducted by means of zero unitarization method (19), whereas aggregation by means of the sum of the products of normalized variables and their attributed weights. The method of zero unitarization is a transformation of diagnostic variables that brings their values to the state of comparability. Its main feature is the adoption of a fixed point of reference, which is the distribution of the variable being subject to normalization (19):

$$R(X_{j})=\underset{i}{\max\;x_{ij}}-\underset{i}{\min\;x_{ij}}$$

Thus, the zero unitarization method takes the following form:

$$Z_{ij}=\frac{x_{ij}-\underset{i}{\min\;x_{ij}}}{\underset{i}{\max\;x_{ij}}-\underset{i}{\min\;x_{ij}}}\;\underset{i}{\max\;x_{ij}}\neq \underset{i}{\min\;x_{ij}}\;X_{j}\in S\text{ for stimulants}$$

$$Z_{ij}=\frac{\underset{i}{\max\;x_{ij}-}x_{ij}}{\underset{i}{\max\;x_{ij}}-\underset{i}{\min\;x_{ij}}}\;\underset{i}{\max\;x_{ij}}\neq \underset{i}{\min\;x_{ij}}\;X_{j}\in D\text{ for destimuli}$$

$$Z_{ij}=\{\begin{array}{cc} \frac{x_{ij}-\underset{i}{\min\;x_{ij}}}{c_{0j}-\underset{i}{\min\;x_{ij}}} & {\text{for x}}_{\text{ij}}\leq {\text{c}}_{0j}\\ \frac{x_{ij}-\underset{i}{\max\;x_{ij}}}{c_{0j}-\underset{i}{\max\;x_{ij}}} & {\text{for x}}_{\text{ij}}>{\text{c}}_{0\text{j}} \end{array}X_{j}\in N\text{ for nominants}$$

Values of the weights attributed to each constituent of the measure were chosen arbitrarily, whereby the key was the significance of a given element for the summary effect of the system functioning. Lower values were also assigned for lower credibility data. A list of weights attributed to specific constituents of the synthetic measure is presented in Table 3. As mentioned, the components of SOM are presented both in nominal values in a given year and in dynamic dimension, which means the rate of change of the value of the given component throughout the given period of time. It was assumed that the higher dynamics defines a higher value of outcome measure. For that reason, all dynamic components have been denominated stimuli (if changing in desired direction), even though the same component presented in a static manner may have another denomination.

**Table 3:** List of weights attributed to specific constituents of the synthetic measure.

	Denomination	Weight
% average number of outpatient medical visits per inhabitant	S	70
% average number of outpatient medical visits per inhabitant (dynamics)	S	70
Bed occupancy	S	80
Bed occupancy (dynamics)	S	80
Average hospitalization length	N	60
Average hospitalization length (dynamics)	S	60
Total number of beds per 100,000 inhabitants	N	90
Total number of beds (dynamics)	S	90
Number of acute beds per 100,000 inhabitants	N	90
Number of beds for short hospitalization (dynamics)	S	90
Beds for short hospitalization as % of all beds	D	90
Beds for short hospitalization as % of all beds (dynamics)	S	90
Life expectancy	S	100
Life expectancy (dynamics)	S	100
Mortality: cardiovascular diseases per 100,000 inhabitants, population 0–64	D	100
Mortality: cardiovascular diseases, population 0–64 (dynamics)	S	100
Mortality: cardiovascular diseases per 100,000 inhabitants, total population	D	95
Mortality: cardiovascular diseases, total population (dynamics)	S	95
Cancer prevalence/100,000 people	D	10
Cancer prevalence (dynamics)	S	10
Mortality: malicious tumor per 100,000 inhabitants	D	100
Mortality: malicious tumor (dynamics)	S	100
Mortality: external causes/100,000 people	D	80
Mortality: external causes (dynamics)	S	80
Incidence: tuberculosis/100,000 inhabitants	D	10
Incidence: tuberculosis (dynamics)	S	10
Mortality: tuberculosis/100,000 inhabitants	D	80
Mortality: tuberculosis (dynamics)	S	80
Mortality: contagious and parasitic diseases/100,000 inhabitants	D	80
Mortality: contagious and parasitic diseases (dynamics)	S	80
Diabetes: % of population	D	10
Diabetes: % of population (dynamics)	S	10
Mortality: diabetes/100,000 inhabitants	D	100
Mortality: diabetes (dynamics)	S	100
Infant mortality/1,000 live birth	D	90
Infant mortality (dynamics)	S	90
Maternal mortality/100,000 live birth	D	90
Maternal mortality (dynamics)	S	90
Alcohol intake per person	D	55
Alcohol intake per person (dynamics)	S	55
Number of smokers as % of mature population	D	60

*D, destimulant; S, stimulant; N, nominants*.

## An Example of using SOM for the Analysis of Health Systems in Central and Eastern European Countries

Countries of Central and Eastern Europe, despite their significant cultural differences, constitute an important research area from the viewpoint of the analysis of health systems effectiveness. It stems from two premises. Firstly, before 1990, these countries shaped their health systems in accordance with the Soviet model, which made them similar in a greater degree than it was in the western part of the continent. Secondly, after the Soviet Union collapsed and the countries regained their sovereignty, they started a process of health system reforms, assuming similar premises and directions for change, showing, however, important differences as to the pace of reformative processes, as well as to individual solutions launched on the grounds of the system in transition (20–22). The above aspects make the differences observed in effects of the functioning of health systems capable of becoming a ground for seeking factors triggering them. This refers to factors having their roots in an accepted reform paradigm, in its course and coherence, and in other areas. In order to determine the value of the SOM index for 21 compared countries, data from the World Health Organization (23) were used, supplemented where necessary with data from databases of World Bank (24) and Organization for Economic Co-operation and Development (25), International Labour Organization (26), and United Nations (27). Such an approach allowed for the comparability of constituent measures of the index.

The index SOM enabled the analysis of health systems in individual countries, both in static and in dynamic approaches, i.e., taking into account changes in the index value in 25 years time span. Static approach to analyze given countries is limited to the scale of 0.6–0.85 points. This has no significance for interpretation, as it is a result of an accepted aggregation and weighting procedure. Interpretation of the coefficient values is possible only in terms of comparison referring individual countries and periods.

**Figure 1:**
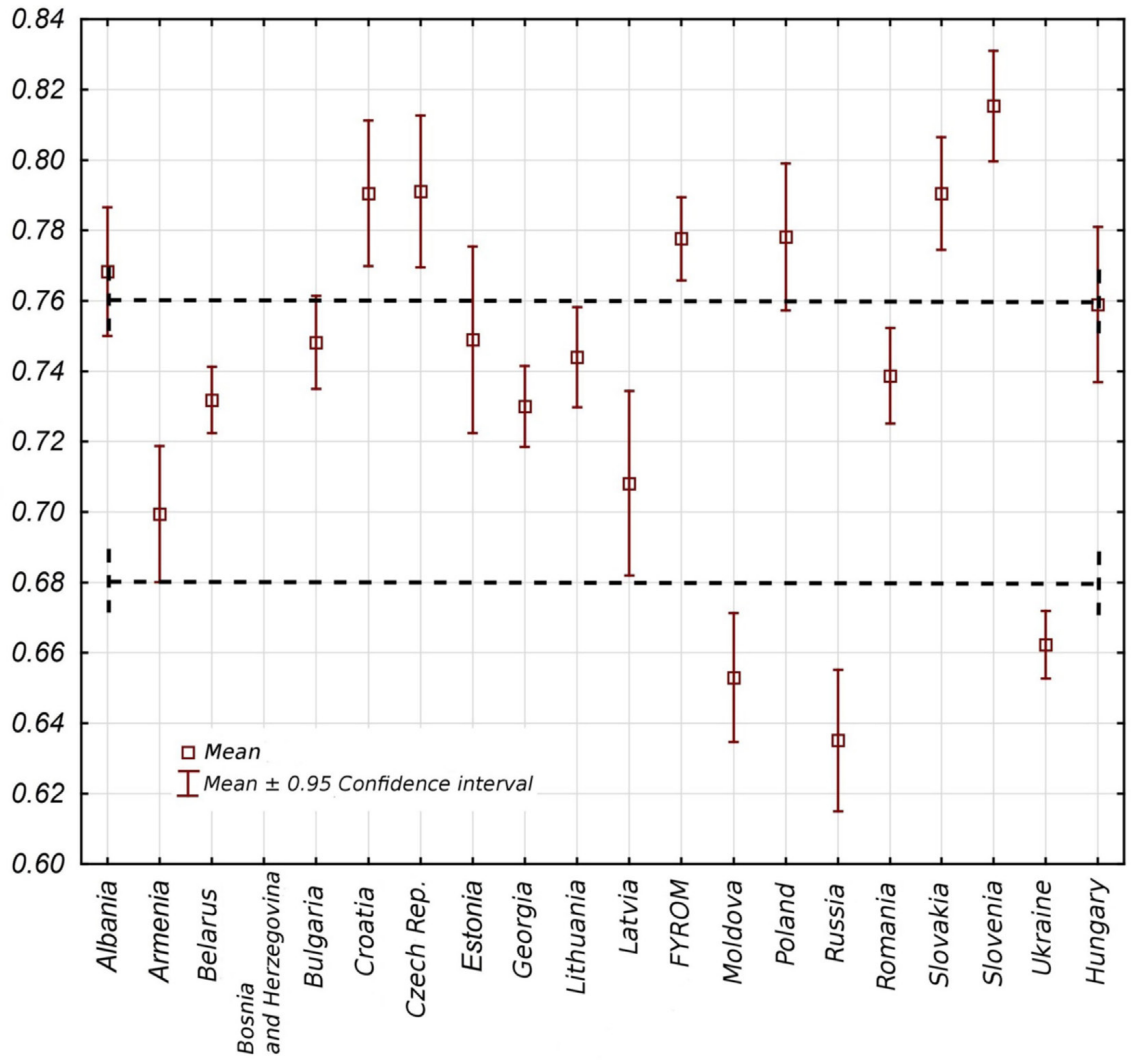
Synthetic outcome measure values for post-communist countries in period 1988–2012.

A comparative study on the average assessment of the effects of health system operation within a group of countries shows in the whole analyzed period 1988–2012 a certain results differentiation; yet it is worth noting that there are little differences in the results of each country. Considering the whole period, a similar level of health system effects can be observed in Slovenia, Croatia, Czech Republic, Slovakia, Poland, Macedonia, and Albania. In the middle group, Hungary, Romania, Latvia, Lithuania, Georgia, Estonia, Bulgaria, Belarus, and Armenia were found. The third group, weakest in terms of achieved effects, was formed by health systems in countries like Ukraine, Moldova, and Russia (Figure 1).

**Figure 2:**
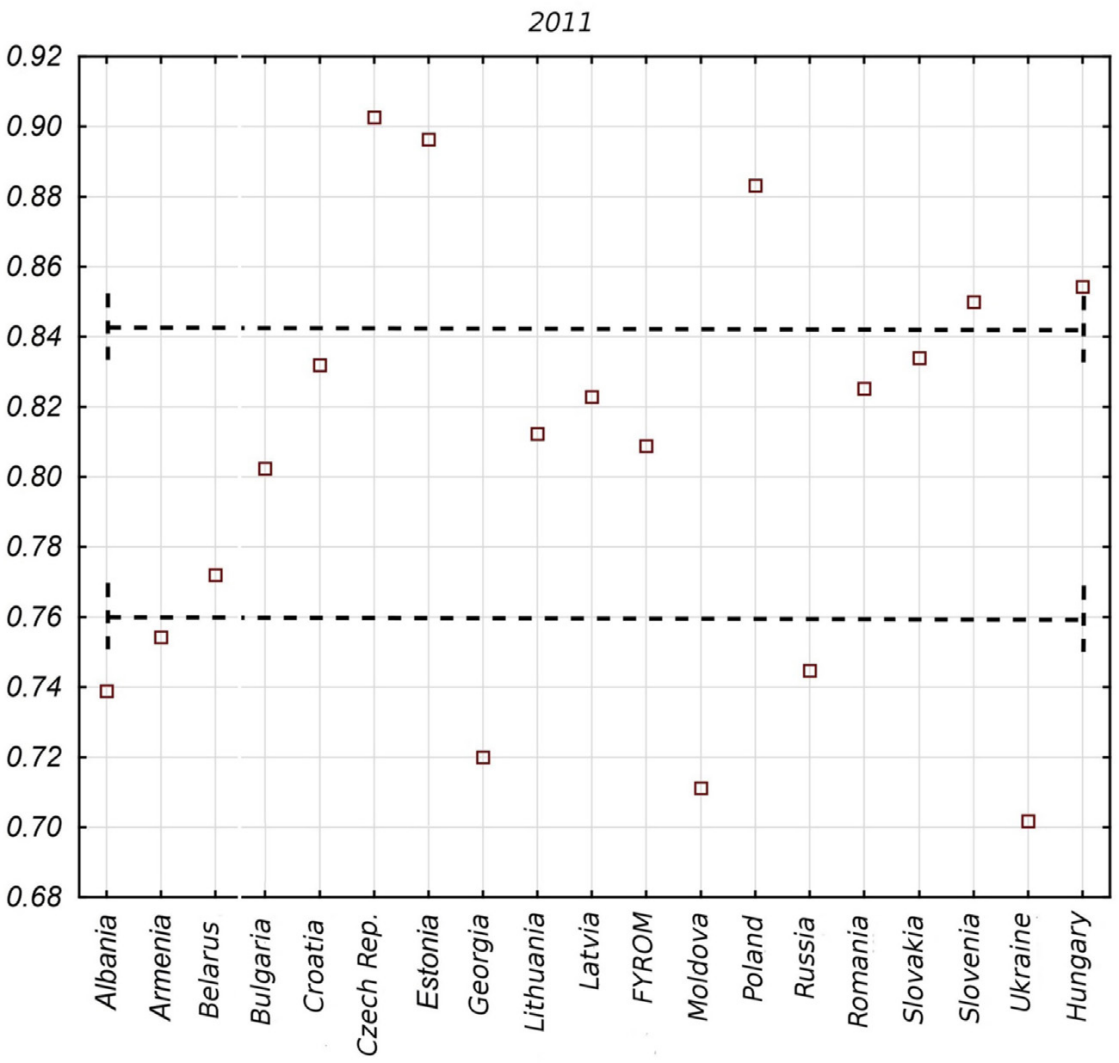
Synthetic outcome measure values in post-communist countries in 2011.

Countries differ in terms of the range of SOM value changes in the whole analyzed period. It is substantial in the case of Estonia and Latvia, relatively important in the case of Poland, Hungary, Czech Republic, Croatia, Russia, and Moldova, and little when it comes to Belarus, Ukraine, Macedonia, Lithuania, and Georgia. This information reveals fluctuation dynamics of the index value in time, yet it does not necessarily mean that in such a dynamic range, an improvement appears in a given country. On the basis of Figure 1, it is not possible to conclude about the direction of changes, which will be a subject of further study. The static value of the SOM index looks differently if the most update complete data are taken into account, which in our case will refer to year 2011. In this situation, a classification of health system effects in Central and Eastern Europe is as follows (Figure 2): the first group of countries with the highest effects consists of Czech Republic, Estonia, Poland, Hungary, and Slovenia. The second group of countries with a moderate level of outcomes includes Slovakia, Croatia, Latvia, Macedonia, Romania, Lithuania, Bulgaria, and Belarus. The third group of countries with the lowest effects in health system functioning includes Ukraine, Russia, Moldova, Georgia, Albania, and Armenia.

**Figure 3:**
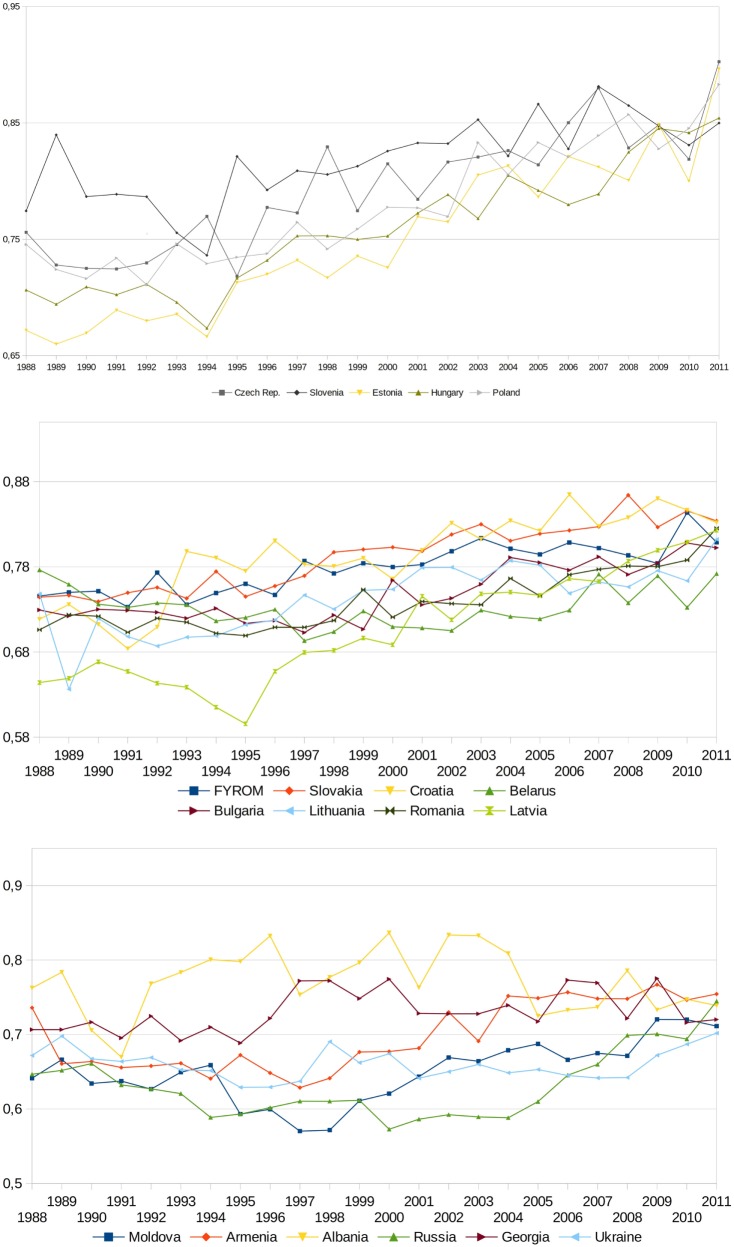
Changes in synthetic outcome measure values for post-communist countries in period 1988–2011.

The presented pictures allow for the indication of a few interesting interpretative perspectives. An important observation is that some of the countries maintain themselves throughout the period constantly at the top of the range in terms of the effects of the functioning of health systems. Furthermore, it may mean that a better initial situation at the process of transformation positively conditions the level of the system at later stages. Simultaneously, it is worth noting that as time progressed, some of the countries moved from one group to another with different effects. In the case of Albania, it was a fall from group one to group three, in the cases of Armenia and Georgia—from the second to the third group, whereas Croatia, Macedonia, and Slovakia—moved from the first group with the highest effects to the second group. On the other hand, Estonia advanced from the second to the first group. Also within individual divisions, countries whose ranking did not change move both directions on the scale. In Figure 3, a progress in terms of SOM value for each country, starting from those with the best result in the final year of study, to those with the worst in the third chart, is presented. Based on this, it is possible to state that transformation processes in individual countries were carried out differently, and besides effects of the health systems, they vary significantly also in terms of the efficacy and intensity of introduced health-care reforms.

## Advantages and Limitations of the Proposed Methodology

The chosen methodology is characterized by a number of advantages when used for the assessment of health system operation. Firstly, it allows for the analysis of the health systems outcomes from a comparative angle, eliminating arbitrariness of pinpointing a model solution as a potential reference point in assessment of the systems. The zero unitarization method applied here sets a value of the measure solely based on an internal relationship of the measure constituents in a group of analyzed systems. Thanks to this, it is possible to conduct a comparative evaluation of employed solutions in order to highlight the best performing ones. The usefulness of such a method reveals itself particularly in the case of similar analyses, where, as already mentioned, in terms of the circumstances and entry situation, a general profile of analyzed countries is rather alike; however, differences between them are easily discernible as regards the intensity of reform processes and individual solutions. Secondly, the measure, with the use of additional statistical tools to establish correlations with elements of the external and internal environment of a health system, allows for conducting analyses of conditions for differences in the effects of health system operation and circumstances for the effectiveness of reform processes. It shall be noted that a catalog of factors to consider is nearly unlimited, and their selection is not conditioned by any means by the selected method of assessing the effectiveness of health systems, which may also be treated as a proof of usefulness of this instrument. Thirdly, the dynamic dimension of the study carried out by its means is also among the benefits of this method. The dynamics can be expressed on two planes. The first plane refers to variability of the value of measure in time, which, besides a simple comparison of measure in a given year or a period, allows for an analysis and comparison of the pace of changes. The second plane involves constituents of the measure in which the nominal value of component measures was included and their dynamic dimension referred to the pace of their change in time. According to the authors, such an attitude is justified by the original aims of a comparative analysis of effectiveness in the systems undergoing transformation processes. Fourthly, an indisputable advantage of this methodology lies in a relative simplicity of construction, foundation on open-access secondary data, and flexibility referred to the scope of a comparative analysis and used constituents of the measure.

Application of the methodology to the comparison of health systems in post-communist countries led to a number of conclusions [more: (28, 29)].

Considering the period between 1988 and 2012, a progress in the effectiveness of health system can be observed in all post-communist countries represented in the study. The study allowed to isolate an exception in this group, namely Albania. In this country, after the initial improvement of the value of the synthetic measure, a deep regression occurred. As a result, Albania is the only example in the group under analysis in which the value of the measure in the last year of the examined period is lower than in its initial moment.

A comparative study allowed for the identification of a group of states with the highest final level of the synthetic measure of the health system effects. This group constitutes Czech Republic, Slovenia, Estonia, Hungary, and Poland. Countries with the lowest value of the measure are Moldova, Armenia, Albania, Russia, Georgia, and Ukraine. A different outlook of the countries is revealed upon considering the dynamics of value changes in the measure—the best result was obtained by Estonia, and in the case of Latvia, located in the intermediate group in terms of the final synthetic measure, a very high dynamics was observed. A systematically decreasing dynamics of value changes can be observed as the groups changed to lower regarding the final value of the measure. The conclusion drawn is that generally a better initial situation of the countries considering the effectiveness of health systems determines a better final situation. This interrelation ceases to exist in the case of dynamics in change. It can be assumed that this element does not depend on initial circumstances but on other factors, such as merit and depth of implemented reforms or economic conditions.

Taking into consideration the moment and intensity of the introduced health-care reforms in the countries under analysis, it is clear that countries which decided to launch complex health-care reforms concurrently achieved a positive outcome with regard to the later growth dynamics of the measurement of health system effects. The value gain of the measure is significantly higher than in the countries which did not execute such reforms or those which did it later.

The methodology, however, is not free from certain limitations, in terms of both its construction and the catalog of the source data used to form it. Firstly, basing on secondary sources entails the danger of distorting analytical results due to errors in source databases. Such errors can occur as a derivative of data falsification, either intended or caused by irregularities in the process of data collection, leading in effect to analytical results that are far from reality. The problem can be solved by a multi-source verification of data; nonetheless, this complicates and delays the analytical procedure to a large extent, limiting at the same time one of the key advantages of the method, namely the simplicity of its formulation resulting from data availability.

In addition, the suggested measure was based on the assumption of complete assessment of the effects of a health system, which results in a comprehensive directory of measures used to develop the instrument. It means that there is a possibility that not every value of used partial measures is a direct result of the impact of the same factors—either those being a derivative of decision processes regarding the transformation of health systems or direct influence of particular external factors. It is also possible that individual determinants of the effects of a health system impact the particular areas to which the measures are employed in an uneven manner. The use of an aggregated instrument does not allow for identification of such differences. Finally, not every measure utilized equally reflects positive and negative trends and phenomena occurring in the health system, for aggregation blurs differences in this matter. It needs to be stressed, as it was already briefly mentioned above that the instrument is flexible and can be modified, with both reduction of a number of partial measures and their clustering resulting in the development of separate measures for different levels of health system functioning. For instance, it is possible to use only measures related to the epidemiological dimension connected to mortality and disease prevalence, or referring only to the health service availability. Under these circumstances, the aggregation of partial measures to create a uniform, synthetic instrument for the assessment within any range and number of partial measures, together with the zero unitarization method employed in the process of comparative analysis, should be treated as key elements of the proposed methodology.

Except for that, partial measures used to develop the synthetic instrument were given weights whose value was set arbitrarily. Although the influence of arbitrariness on the final effect of the comparative study in which synthetic measure was used for the assessment of the systems is little when weights are of equal value in every country, it is a factor potentially restricting the instrument usability. It is so particularly while broadening the scope of the analysis and reaching out of a relatively homogeneous group of juxtaposed countries. If potential reduction of the number of measures in the synthetic instrument and introduction of the aggregation procedure for clustered measures of more homogeneous internal structure take place, the scale of the impact of the weights arbitrariness will also decrease. Nevertheless, bearing in mind that the presented methodology is in its preliminary stage of development, it must be assumed that its evolution should involve objectivization of weights assigned to particular measures on the basis of external criteria, describing their actual value for the purpose of health system effectiveness assessment.

Finally, the study of health systems carried out by means of the proposed instrument overlooks certain important elements of their profile, such as measurement of effectiveness understood as a relationship of the output to the input. The suggested instrument enables performing similar analysis assuming other assessment criteria in the form of the measure value to the input ratio. Moreover, some of the assessed health system dimensions from the catalog, like those referring to equality in access to health care or quality of medical services, were omitted. Also in this case, the presented methodology allows for the use of partial measures relating to those dimensions, provided that objective and comparable measures of those aspects in the systems of compared countries are employed.

## Author Contributions

PR conceived the study, performed a literature study, contributed to paper draft, and prepared the final version of the manuscript. KK contributed to literature study and prepared the draft of the paper. MS-Ś contributed to the draft of the paper. TH contributed to the draft of the paper. AS contributed to study methodology and literature study.

## Conflict of Interest Statement

The authors declare that the research was conducted in the absence of any commercial or financial relationships that could be construed as a potential conflict of interest.
